# Low-toxic and organic solvent-free isolation of RNA

**DOI:** 10.1371/journal.pone.0345312

**Published:** 2026-04-15

**Authors:** Romy Böttcher, Thomas Fröhlich, Klaus Förstemann

**Affiliations:** Gene Center and Dept. of Biochemistry, Ludwig-Maximilians-Universität München, Feodor-Lynen-Strasse, München, Germany; Anhui University of Chinese Medicine, CHINA

## Abstract

RNA is the central molecule that connects genetic makeup to the function of living cells and organisms. Since genes will also cease to be transcribed, RNA must decay so that the cellular RNA-pool can reflect the current transcriptional state. Undesired degradation is therefore a major challenge when RNA is isolated from living cells, tissues or organisms. Strongly protein-denaturing conditions, often involving toxic organic solvents, are usually employed to quickly inactivate RNases prior to separating nucleic acids from protein. We propose to combine high concentrations of urea, a compound used in fertilizers, with SDS as a non-volatile, low toxicity denaturant. The use of urea and SDS has previously been combined with organic solvent extraction, chaotropic salt-mediated column purification or selective precipitation of long RNA with LiCl. Here, we demonstrate that – akin to the classic alkaline lysis protocol for plasmid preparation – addition of potassium acetate forms insoluble potassium dodecyl sulfate, which can be removed along with denatured proteins and part of the genomic DNA by centrifugation. The RNA is then precipitated with isopropanol, recovering also small non-coding RNAs. We demonstrate that these somewhat crude RNA preparations are nonetheless sufficiently clean to be quantified via classic UV absorption measurements, stable over the time-course of a typical molecular biology reaction and do not harbor inhibitors of reverse transcriptase. While it seems unlikely that we are the first to conceive this simple strategy, we are not aware of any corresponding prior publication.

## Introduction

Ribonucleic acid (RNA) was the first genetic material during evolution and is the immediate product of genetic activity. It is therefore not surprising that there is no shortage of protocols for the isolation of RNA from biological sources [1–7]. Due to the presence of a 2’-OH group on the ribose, RNA is inherently more prone to chemical fragmentation than DNA. In cells, many RNA species are rapidly turning over or even actively targeted for degradation, for example in the context of pathogen defense. The prerequisite for isolating intact RNA is therefore a rapid, ideally instantaneous, inactivation of all RNA degrading processes upon cell or tissue lysis.

To achieve this goal, RNA isolation is often carried out under harsh protein-denaturing conditions while keeping chemical hydrolysis to a minimum. Many protocols employ guanidinium salts and phenol/chloroform extraction during lysis [2]; the concept of treating cells with organic solvents can even be traced back to Miescher’s discovery of nucleic acids [8]. This is then followed by removal of the denatured proteins via centrifugation where denatured proteins will accumulate at the phase boundary (often called interphase) of the two-phase system. If an acidic pH is chosen, DNA will preferentially partition into the phenolic phase whereas RNA remains in the aqueous supernatant. While certainly versatile and successful, this strategy requires chemical fume hoods to safely work with volatile substances and generates a certain amount of toxic waste, in particular halogenated organic solvents.

An alternative approach is to perform the lysis step with high concentrations of guanidinium thiocyanate, then purify the nucleic acids by adsorption to a silica matrix (e.g., spin column, magnetic beads). This allows for efficient washing and elution steps, but the procedure is less well suited for small RNAs (< 200 nt). At least some protocols require the addition of β-mercapto-ethanol, a toxic and volatile compound with an unpleasant smell that must be used in a fume hood. While a modification of the binding conditions can retain the small RNA fraction, this can also lead to more genomic DNA in the preparation; pre-fractionation via acidic phenol extraction is generating more toxic waste as a consequence.

All this is not problematic in a normal laboratory setting, but it can impose limitations for, e.g., field work or laboratories in remote locations (e.g., lengthy delivery of solvents as dangerous goods, problematic waste disposal). Samples collected in the field can be conserved by non-toxic preservatives such as concentrated ammonium sulfate solutions (e.g. RNAlater) until extraction. Nonetheless, due to a limiting number of fume hoods this can become cumbersome for the team upon the return. Efforts to conceive less toxic approaches, such as the ammonium trichloroacetate / SDS method [9], have thus been made.

### Are there alternative solutions for purifying RNA without the use of organic solvents or toxic substances, only using common molecular biology reagents?

High concentrations of urea, which is an important fertilizer for agriculture and not classified as toxic compound, have previously been employed to denature proteins during RNA isolation [10–12]. Usually, this has been followed up by extraction with phenol/chloroform to separate protein from RNA. If only the larger RNA species are needed, selective precipitation of only the RNA with LiCl can be used instead [13]. In this manuscript, we are proposing a combination of concentrated urea with sodium dodecyl sulfate (SDS) to lyse cells or tissues under strongly denaturing conditions, using only reagents that are generally available in a molecular biology laboratory. The proteins and in part also the long, entangled genomic DNA are then precipitated along with the detergent via addition of potassium acetate, just as in the classic alkaline lysis protocol for bacterial plasmid DNA preparations. The remaining nucleic acids are subsequently recovered by addition of isopropanol, retaining also smaller RNAs. While the alcoholic supernatants and the precipitated potassium dodecyl sulfate still should be collected as special waste, their hazards are considerably lower than phenol/chloroform and thus easier to mitigate.

Interestingly, for very small amounts of starting material such as single-cell analyses, cell lysis with mild detergents followed by cDNA generation without any purification (“direct lysis”, also commercially available) has emerged as a valid strategy [14,15]. The assumption is that the target RNA molecules exist as ribonucleoprotein complexes in the cells and the associated proteins provide some protection from degradation by RNases. When the sample amounts are severely limiting, the direct cDNA synthesis strategy appears superior to the inevitable losses that occur during RNA purifications steps, e.g., due to adsorption to plastic surfaces. Yet, for RNA extraction from larger cell numbers, complex tissues or whole animals such as fruit flies, the isolation and purification of RNA under protein-denaturing conditions remains the gold-standard strategy. This is even essential if the RNA needs to be further fractionated (e.g. polyA-tail or size-selection) before analysis.

### Limitations of urea-based protocols

It is important to keep in mind that urea will also denature nucleic acid duplexes (as in DNA and RNA secondary structures); this is employed in denaturing acrylamide-urea gel electrophoresis of nucleic acids (e.g., sequencing gels). Thus, if the experiment requires preservation of structures or complexes of multiple nucleic acid molecules, our protocol is *not* a valid option.

When urea is dissolved in water, a dissociation reaction will take place and generate cyanate ions in low millimolar concentrations once the chemical equilibrium is reached after several days at room temperature [16]. As a result, carbamoylation of lysine side chains and the amino-terminus in proteins will occur; this may even be beneficial by inactivating RNase enzymes [17]. However, it is at least conceivable that cyanate also reacts to with functional groups on nucleic acid bases during the isolation; note that this has not been perceived as an issue during electrophoresis in acrylamide/urea sequencing gels. If this is of concern (e.g., study of RNA modifications), the extraction buffer should be prepared by freshly dissolving the urea. Furthermore, inclusion of ammonium carbonate in the extraction buffer at 1 M concentration may reduce the extent of the protein carbamoylation reaction by favoring the urea state in the chemical equilibrium [18]. While cyanate is biodegradable [19], it may accumulate to more than negligible levels in urea-containing buffers during storage. We therefore recommend to include ammonium carbonate in our lysis buffer, but the protocol also works well without it.

We implemented the Urea/SDS strategy in our laboratory in an effort to avoid “traffic jams” at our fume-hood equipped for RNA isolation. It is highly unlikely that we are the first to imagine this approach, but we are not aware of a corresponding publication. While the protocol certainly cannot replace guanidinium or phenol-based protocols for all applications, we find it yields RNA of sufficient quality and stability for many of our needs. The procedure is neither faster nor does it require fewer pipetting steps than a Trizol-prep, but it can be performed on any of our laboratory benches with readily available reagents. This provides a very welcome flexibility for the collaborators.

Link to the Protocols.io Platform: DOI: dx.doi.org/10.17504/protocols.io.n92ld6deog5b/v1

## Results and discussion

**Fig 1:**
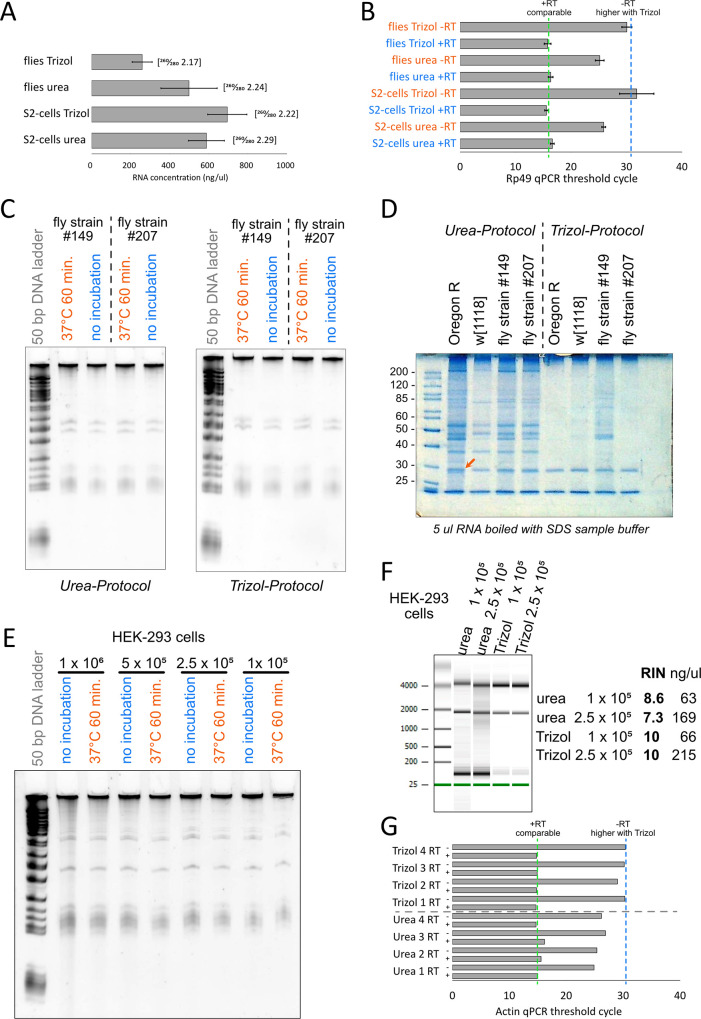
Evaluation of the Urea/SDS based RNA extraction protocol. A) Comparison of RNA yields between the Urea/SDS and Trizol-based extraction protocols. B) Evaluation by qRT-PCR of mRNA content (+RT) and genomic DNA contamination (-RT) for the Urea/SDS and Trizol protocols with *Drosophila* samples; cDNA was generated with the RevertAid kit (Thermo) using random hexamer primers, qPCR was performed with the DyNAmo Flash SybrGreen qPCR kit (Thermo) using *rp49* specific primers 5’-ACAATCTCCTTGCGCTTCTT-3’ and 5’-ATCGGTTACGGATCGAACA-3’. C) Comparison of RNA stability for fly samples processed with the Urea/SDS (left) and Trizol (right) protocols; 100 ng of RNA was incubated as indicated and then separated on an 8% acrylamide-urea gel; the region visualized corresponds to roughly the tRNA size range and longer transcripts; note that the marker is a 50 bp DNA ladder, which is not ideal for size estimation on denaturing gels and for RNA samples, hence we refrained from annotating the size; D) Protein content of the RNA samples assessed by separating 5 ul of the final RNA solution on SDS-PAGE and staining the proteins with colloidal Coomassie-Blue. The band that was excised and analyzed by mass spectrometry is indicated with a red arrow. E) Comparison of RNA stability for HEK293 samples processed with the Urea/SDS protocol; 100 ng of RNA was incubated as indicated and then separated on an 8% acrylamide-urea gel; the region visualized corresponds to roughly the tRNA size range and longer transcripts; the number of cells used as starting material is indicated above. F) Comparison of RNA yield and quality obtained with the Urea/SDS protocol and Trizol using the indicated number of HEK293 cells as starting material. Left: Image output from a BioAnalyzer run; Right: Table with the corresponding RNA integrity numbers (RIN) as well as the concentrations determined in the BioAnalyzer run. G) Evaluation by qRT-PCR of mRNA content (+RT) and genomic DNA contamination (-RT) for the Urea/SDS and Trizol protocols with HEK293 samples; cDNA was generated with the RevertAid kit (Thermo) using random hexamer primers, qPCR was performed with the DyNAmo Flash SybrGreen qPCR kit (Thermo) using *actin* specific primers 5’-GGCATCCTCACCCTGAAGTA-3’ and 5’-GTCAGGCAGCTCGTAGCTCT-3’. For each sample, the top bar represents the -RT control and the bottom bar the + RT reaction. The green dashed line indicates the c_T_-value for the + RT-reaction with Trizol while the blue dashed line indicates the c_T_-value for the corresponding -RT reactions.

We first compared the RNA yield between the urea and the Trizol method using *Drosophila* S2-cells and flies (Fig 1A, 4 parallel RNA preparations). The yield appears to be comparable for S2-cells and even somewhat better for the urea-protocol when using flies (standard deviation was calculated from parallel preps). The latter needs to be verified on a larger set of samples processed on different days, though. Nonetheless, we conclude that for the *Drosophila* samples the urea protocol produces yields that are comparable to the established Trizol-method.

We then compared the mRNA and genomic DNA content for 500 ng of total RNA by quantitative RT-PCR (Fig 1B, n = 4). We synthesized cDNA with the help of random hexamer primers, then amplified the *rp49* sequence (a housekeeping-gene that is frequently used for normalization). The mRNA content is comparable between the protocols (+RT samples), but the urea-protocol results in a lower threshold cycle (~6 cycles difference) for the -RT samples, indicating a substantially higher about of contaminating genomic DNA. This will require DNase treatment when low abundance transcripts are analyzed, but for the abundant *rp49* cDNA there was still a difference of ~9 cycles between the + RT and the -RT samples for the urea-based preparation. Thus, while the DNA removal is certainly not perfect, it can be sufficient for a range of needs.

One of the main virtues of a good RNA preparation is to be free of contaminating RNases. We tested this by incubating our RNA samples for 60 minutes at 37°C to simulate a typical molecular biology reaction, then loaded the samples along with controls kept on ice on an 8% acrylamide-urea gel (Fig 1C). Judging by the sharpness of the visible bands (likely tRNAs etc.), no obvious RNA degradation occurred during this incubation. We also compared cDNA synthesized after the 60 minutes 37°C incubation with the help of our qPCR assaying the *rp49* transcript. Again, there was no difference between two control samples (Ct-values of 15.16 and 15.52) and the same samples incubated for 60 minutes at 37°C (Ct-values of 15.21 and 15.12). We also did not observe any DNase activity, since the -RT samples were also equal before (Ct-values of 25.10 and 26.21) and after the incubation (Ct-values of 24.80 and 25.91). We conclude that the RNA obtained with the urea-protocol is free of contaminating nuclease activities.

How well are all the other proteins removed? A simple potassium dodecyl sulfate co-precipitation strategy is likely not as efficient as the extraction with an organic solvent. Indeed, when we boiled 5 µl of total RNA (~2.5–3.5 µg) in SDS sample buffer, loaded the samples on an SDS-PAGE and stained this with colloidal Coomassie, we saw clearly more protein bands with the urea-protocol. Interestingly, though, even the Trizol-samples were not entirely free of protein either; in particular, a prominent band was visible at a MW of just under 30 kDa (fly samples shown in Fig 1D, similar results were observed with S2-cells). We subjected this band (from RNA extracted from flies) to mass spectrometry analysis and identified myosin light chain 2 (CG2184, 222 amino acids, MW 23,7 kDa, pI 4.67), ATP synthase subunit gamma (CG7610, 297 amino acids, MW 32,9 kDa, pI 9,29), ADP-ATP carrier Protein *sesB* (CG1299, 299 or 312 amino acids, MW 34,2 kDa or 32,9 kDa, pI 9,82 / 9,80) and *Drosophila* Prohibitin *Phb1* (CG10691, 276 amino acids, MW 30,4 kDa, pI 5,5) as major components. The first protein is abundant in flight muscle and the latter three are localized in or at the inner mitochondrial membrane; since mitochondria are abundant in flight muscle and other cells, this contamination may simply reflect the original abundance of the proteins and perhaps their solubilization into small detergent micelles, which were not completely removed by the centrifugation step.

We verified that our protocol can be applied to cultured mammalian cells as well and subjected HEK-293T cells to our procedure. We obtained RNA as expected and the incubation at 37°C indicated that no degradation occurred during this time (Fig 1E). These cells are much larger than Drosophila S2-cells, hence the resuspension becomes difficult for identical cell numbers and thus extraction / inactivation may not be as efficient. The RNA quality is acceptable for 1x10^5^ and 2.5x10^5^ cells extracted with 100 ul of our buffer (Fig 1F). We also tested higher amounts but saw only a marginal increase in RNA yield when more than 5x10^5^ cells were extracted (1x10^5^ cells: 42 ng/ul; 2.5x10^5^ cells: 76 ng/ul; 5x10^5^ cells: 117 ng/ul; 1x10^6^ cells: 124 ng/ul). We thus recommend that 5x10^5^ human cells should not be exceeded per 100 µl of extraction buffer. The isolated RNA could be reverse transcribed and yielded comparable threshold cycles to parallel Trizol-Preps; again, the -RT reactions indicate a higher background of genomic DNA in the urea-based RNA preparations (Fig 1G).

In summary, we propose a simple and low-toxicity protocol to isolate RNA from cells and tissues. For the latter, it is important that the tissue can be mechanically disintegrated rapidly so that the extraction solution can act to lyse cells and denature RNA degrading enzymes. There is an upper limit of how many cells can be isolated per 100 µl of our extraction buffer, presumably because at some point the viscosity increases too much due to release of genomic DNA. We advise against strong shearing forces, however, as this may lead to a higher background of DNA in the final RNA sample.

In our hands, the final RNA solution is as stable as a standard Trizol-based RNA prep. Naturally, it is free of phenol but the amount of protein and DNA contamination is higher. None of this seems to reduce the efficiency of reverse transcription, though. It is in principle possible to further purify the RNA with a silica-based solid phase extraction protocol (e.g., spin-column) instead of isopropanol precipitation. In this case, the salt concentration of the KOAc in the precipitation solution must be increased to 3 M and the ethanol concentration raised to 40% in the supernatant before loading onto the column, followed by one wash with 3 M NaAc and another wash with 75% ethanol prior to elution with RNase-free water [20]. It appears more straightforward, however, to stick with the established guanidinium-based methods for such RNA preparations. Our RNA extraction solution is also not a replacement for RNA-preserving fixatives such as RNAlater: When we resuspended *Drosophila* cells in our extraction buffer and left the sample over night at room temperature, the recovered RNA was in part degraded (data not shown). We thus recommend to carry out the protocol without lengthy interruptions.

To summarize, we present a protocol that extracts RNA without using organic solvents and no chemical fume hood is necessary. In our laboratory, the protocol is predominantly employed when RNA preps need to be done for screening cell cultures or fly strains by RT-PCR. It may be a convenient alternative for those who wish to reduce the number of active waste-streams in the lab (no chloroform) or if the available fume hoods are limiting (no solvents, no mercaptan). We also note that it may be cheaper than using commercial silica-based column kits. Akin to the established RNA extraction protocols, we assume that our approach must be modified for challenging samples with strong cell walls, secondary metabolites or very high RNase content.

## Supporting information

S1 File Protocol from protocols.io. (PDF)

S2 File Collection of original images. (PDF)

S1 Table Original values of RNA yields used for calculation and graphs. (XLSX)

S2 Table Original qPCR Data S2 cells. (XLSX)

S3 Table Original qPCR Data flies. (XLSX)

S4 Table Original qPCR Data HEK cells. (XLSX)

S5 Table Original Data mass Spectrometry. (XLSX)
