# Supplementary material for: Low-toxic and organic solvent-free isolation of RNA
Source: PLoS One. 2026 Apr 15;21(4):e0345312. doi: 10.1371/journal.pone.0345312 (PMC13082646; doi:10.1371/journal.pone.0345312)
Supplement: S1 File — (PDF) [file pone.0345312.s001.pdf]

Feb 07, 2026

UREA-SDS quick RNA isolation

DOI
https://dx.doi.org/10.17504/protocols.io.n92ld6deog5b/v1

Klaus Förstemann[1]
[1]University of Munich (LMU)

Klaus Förstemann

Create & collaborate more with a free account

Edit and publish protocols, collaborate in communities, share insights through comments, and track progress with run records.

Create free account

OPEN ACCESS

DOI: https://dx.doi.org/10.17504/protocols.io.n92ld6deog5b/v1

Protocol Citation: Klaus Förstemann 2026. UREA-SDS quick RNA isolation. **protocols.io**
https://dx.doi.org/10.17504/protocols.io.n92ld6deog5b/v1

License: This is an open access protocol distributed under the terms of the **Creative Commons Attribution License**, which permits unrestricted use, distribution, and reproduction in any medium, provided the original author and source are credited

Protocol status: Working
We use this protocol and it's working.

Created: November 21, 2025

Last Modified: February 07, 2026

Protocol Integer ID: 233159

Keywords: sds quick rna isolation rna, rna preparation, selective precipitation of long rna, quick rna isolation rna, nucleic acids from protein, separating nucleic acid, rna, cellular rna, high concentrations of urea, long rna, rnase, use of urea, denatured protein, fertilizer, transcriptase, classic alkaline lysis protocol, akin to the classic alkaline lysis protocol, genomic dna by centrifugation, involving toxic organic solvent, toxic organic solvent, protein, current transcription state, organic solvent extraction, urea, molecular biology reaction, insoluble potassium dodecyl

Funders Acknowledgements:
DFG
Grant ID: 419138605

Abstract

RNA is the central molecule that connects genetic makeup to the function of living cells and organisms. Since genes will also cease to be transcribed, RNA must turn over so that the cellular RNA-pool can reflect the current transcription state. Undesired degradation is therefore a major challenge when RNA is isolated from living cells, tissues or organisms. Strongly protein-denaturing conditions, often involving toxic organic solvents, are usually employed to quickly inactivate RNases prior to separating nucleic acids from protein. We propose to combine high concentrations of urea, a compound used in fertilizers, with SDS as a non-volatile, low toxicity denaturant. The use of urea and SDS has previously been combined with organic solvent extraction, chaotropic salt-mediated column purification or selective precipitation of long RNA with LiCl. Here, we demonstrate that - akin to the classic alkaline lysis protocol for plasmid preparation - addition of potassium acetate forms insoluble potassium dodecyl sulfate, which can be removed along with denatured proteins and part of the genomic DNA by centrifugation. The RNA is then precipitated with isopropanol, recovering also small non-coding RNAs. We demonstrate that these somewhat crude RNA preparations are nonetheless sufficiently clean to be quantified via classic UV absorption measurements, stable over the time-course of a typical molecular biology reaction and do not harbor inhibitors of reverse transcriptases. While it seems unlikely that we are the first to conceive this simple strategy, we are not aware of any corresponding prior publication.

Guidelines

Starting material:
- in our tests, up to 2 million Drosophila S2-cells can be extracted with the volumes given here.
- we suggest using no more than 4 flies with the indicated volumes, otherwise the final pellet will be difficult to dissolve.
- for HEK293 cells, which are much larger, we found that 500'000 cells should not be exceeded for the volumes indicated here.

Materials

Extraction buffer (for 10 ml):
- weigh 4.8 g of urea and transfer the powder into a 15 ml Falcon.
- add 0.79 g of ammonium hydrogen carbonate (optional but recommended)
- dissolve the salts in a small amount of water; start by adding 1 ml, then add more and more water as needed until the final volume is just below 9 ml (solution gets very cold; a small amount of urea may remain insoluble)
- add 500 ul of the 1M buffer stock (Tris-HCl pH 8.8 – hint: same as for pouring protein gels)
- add 500 ul of 10% SDS solution
- add 200 ul of 0.5 M EDTA (pH 8.0)

the final volume will be about 10 ml.

Precipitation solution (for 30 ml):
- weigh 2.95 g of KOAc
- dissolve in 20 ml of water
- add 6 ml of 100% Ethanol (final conc. will be 20%)
- adjust the pH to 7.4 with KOH / acetic acid
- adjust the final volume to 30 ml

Troubleshooting

Safety warnings

Attention:
Neither 8M urea nor 0.5% SDS is "gentle"; they will denature the proteins of your skin and eyes exactly like the ones in your sample. Use appropriate safety measures!

Before start

To preserve the integrity of RNA, Lysis must occur under strongly denaturing conditions. This protocol avoids the use of Phenol/Chloroform and can therefore be carried out at the bench (no need for a fume hood). The approach combines 8M urea with 0.5% SDS and 10 mM EDTA as extraction buffer to inactivate degradation enzymes (RNases). Tris-HCl is buffering the pH, we use 50 mM final concentration and a moderately alkaline pH of 8.8 (optimized for RNA extraction from cultured *Drosophila* cells).
This is, in fact, the very same buffer stock as used for pouring the separating gel for an SDS-Page in our lab.

Comments:
Urea denatures proteins as well as nucleic acids (i.e. makes them single-stranded).
SDS also denatures proteins and helps to remove them as a co-precipitate with potassium dodecyl sulfate. The EDTA chelates bivalent metal ions, which might otherwise promote auto-hydrolysis of RNA during the 65°C incubation step. The ammonium hydrogen carbonate disfavors the dissociation of urea and the resulting non-specific carbamoylation.
Ethanol is added to the precipitation solution both as a preservative and because it increases the efficiency of nucleic acid precipitation in the subsequent step, in particular for the small RNAs. Note that potassium chloride cannot substitute the potassium acetate as it will partially precipitate in 20% Ethanol.

RNA isolation

1 - harvest cells by briefly centrifuging, take off the cell culture supernatant.

2 - resuspend the cell pellet in 100 ul of extraction buffer (see above) by slowly pipetting up & down 2 times (or more until resuspension is complete) to mix GENTLY. Try to avoid shearing the DNA, as this will increase your DNA background.

3 - for flies: sort into microcentrifuge tube, chill on ice for anesthesia, add 100 ul of extraction buffer and crush flies thoroughly with a 1 ml pipette tip.

4 - incubate at 65°C for 10 minutes to increase the extraction efficiency; this will likely denature any nucleic acid duplexes in concert with the high urea concentration.

5 (all solutions added from this step on should be free of RNases as much as possible)

6 - add 200 ul of the precipitation solution and mix gently by inverting the tube several times; incubate 5 min. on ice (a precipitate will form – this is the same approach as for a plasmid mini-prep).

7 - centrifuge 10 minutes at full speed in a table-top centrifuge (as in a plasmid mini-prep).

8 - take off the supernatant (should be ~300 ul at this point; leave a bit on the pellet to avoid aspirating any of the precipitate or genomic DNA that “lingers” above it) and mix this well with an equal volume of isopropanol.

9 - Let stand at room temperature for 5 minutes. If only a small amount of cells was used, including 1 ul RNA-grade glycogen as a carrier before the addition of isopropanol may increase the yield and result in a more easily visible pellet.

10 - centrifuge at top speed in a table-top centrifuge for 10 minutes; discard the supernatant.

11 - wash the pellet (this can also be just a smear along the tube wall) 2x with 100 ul of 70% EtOH (= add, mix, spin 30 seconds full speed, remove supernatant).

12 Note: If you do not want to use the RNA right away, it is best to store it as a pellet under 70% EtOH in a -20°C freezer.

13 - dry the pellet briefly but completely.

14 - dissolve the pellet in e.g. 20 ul of water (flick, mix, wait 30 sec. , repeat, spin just enough to collect the solution at the bottom of the tube).
