# Supplementary material for: Low-toxic and organic solvent-free isolation of RNA
Source: PLoS One. 2026 Apr 15;21(4):e0345312. doi: 10.1371/journal.pone.0345312 (PMC13082646; doi:10.1371/journal.pone.0345312)
Supplement: S2 File — (PDF) [file pone.0345312.s002.pdf]

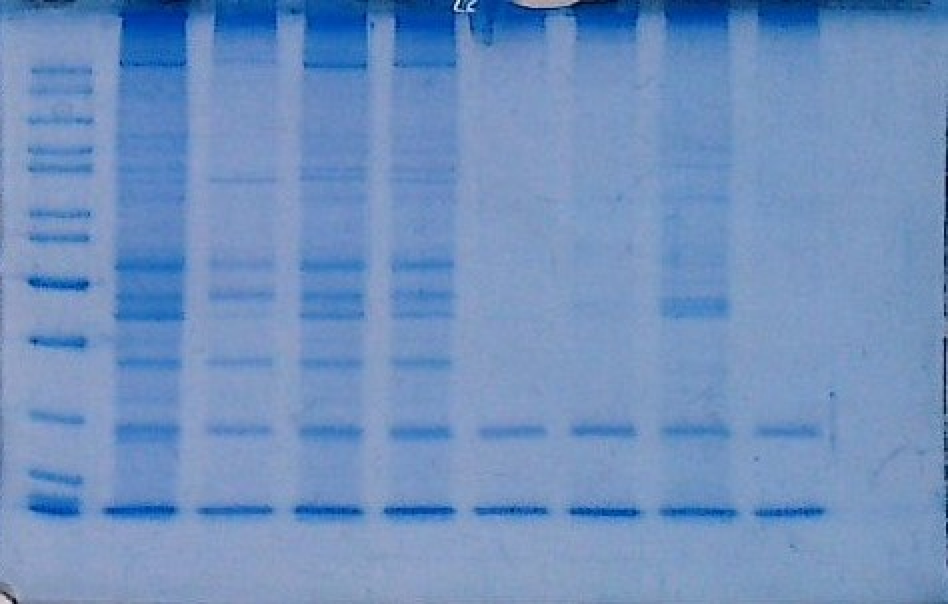

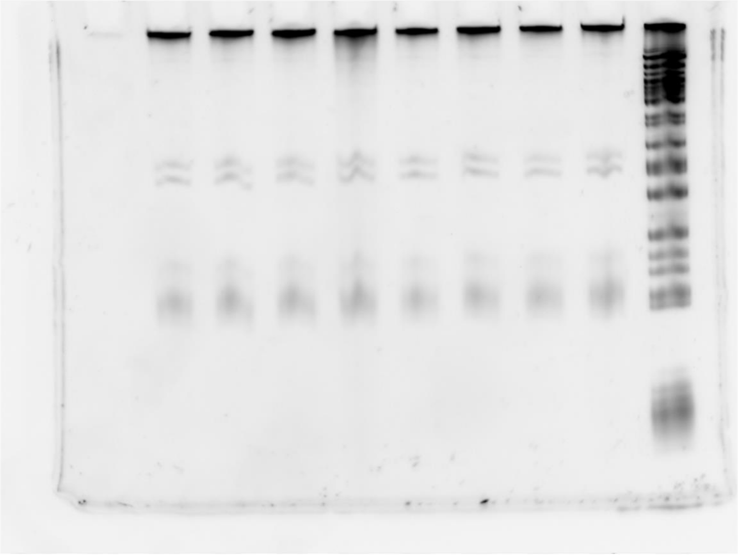

Name: 2023-03-27_17-27-39
User: Default
Exp. Date: 2023-03-27 17:27:39
Shutter: 510 ms
Gain: 0.0

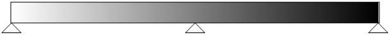

Range: 0.0% - 100.0%, Gamma: 1.000

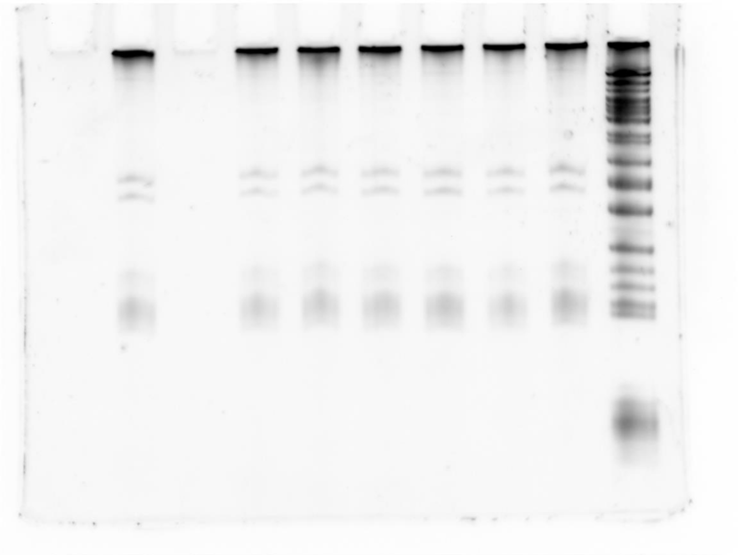

Name: 2023-03-27_17-26-13
User: Default
Exp. Date: 2023-03-27 17:26:13
Shutter: 510 ms
Gain: 0.0

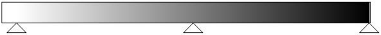

Range: 3.9% - 100.0%, Gamma: 1.000

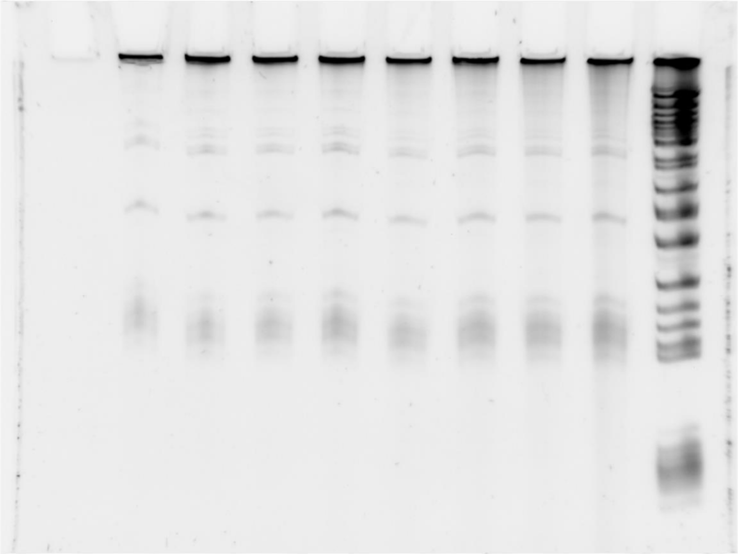

Name: 2023-04-13_18-52-04
User: Default
Exp. Date: 2023-04-13 18:52:04
Shutter: 590 ms
Gain: 0.0

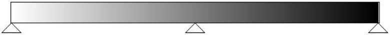

Range: 0.0% - 100.0%, Gamma: 1.000

Assay Class: Eukaryote Total RNA Nano
Data Path: C:\...Eukaryote Total RNA Nano_DE72901994_2026-01-21_15-05-37.xad

Created: 21-Jan-26 3:05:37 PM
Modified: 21-Jan-26 5:35:29 PM

Electrophoresis File Run Summary

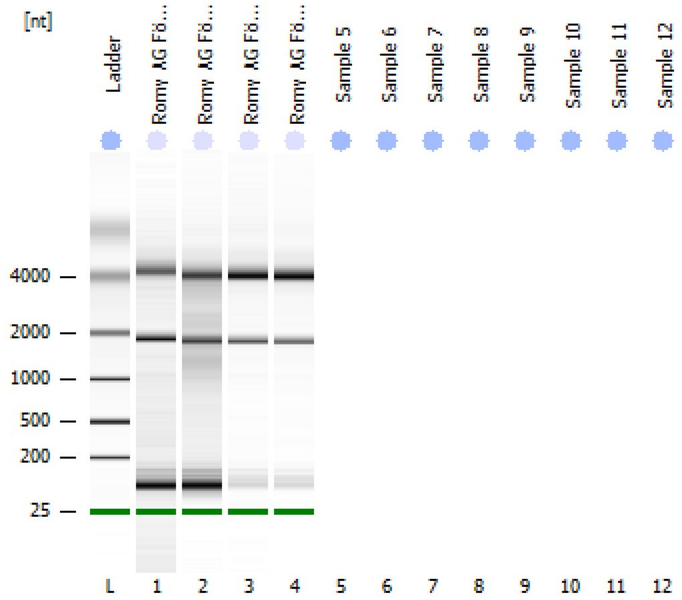

Instrument Information:

Instrument Name: DE72901994 Firmware: C.01.069

Serial#: DE72901994 Type: G2939A

Assay Information:

Assay Origin Path: C:\Program Files (x86)\Agilent\2100 bioanalyzer\2100 expert\assays\RNA\Eukaryote Total RNA Nano Series II.xsy

Assay Class: Eukaryote Total RNA Nano

Version: 2.6

Assay Comments: Total RNA Analysis ng sensitivity (Eukaryote)

© Copyright 2003 - 2009 Agilent Technologies, Inc.

Chip Information:

Chip Lot #:

Reagent Kit Lot #: 2302

Chip Comments:

Romy AG Förstemann 1 origRNA

RIN: 8.60

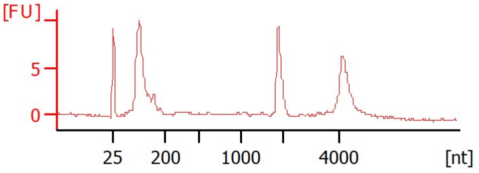

Romy AG Förstemann 2 origRNA

RIN: 7.30

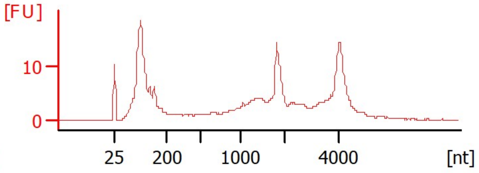

Romy AG Förstemann 3 origRNA

RIN:10

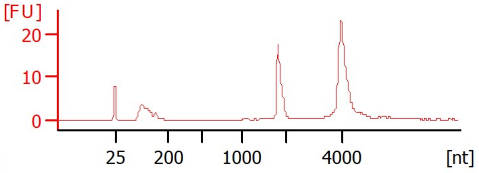

Romy AG Förstemann 4 origRNA

RIN:10

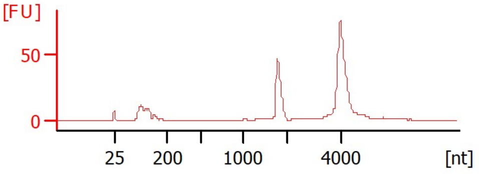

© Copyright 2003 - 2018 Agilent, Inc.

Assay Class:	Eukaryote Total RNA Nano	Created:	21-Jan-26 3:05:37 PM
Data Path:	C:\...Eukaryote Total RNA Nano_DE72901994_2026-01-21_15-05-37.xad	Modified:	21-Jan-26 5:35:29 PM

Electrophoresis File Run Summary (Chip Summary)

Sample Name	Sample Comment	Status	Result Label	Result Color
Romy AG Förstemann 1 origRNA		✔	RIN: 8.60	
Romy AG Förstemann 2 origRNA		✔	RIN: 7.30	
Romy AG Förstemann 3 origRNA		✔	RIN:10	
Romy AG Förstemann 4 origRNA		✔	RIN:10	
Sample 5				
Sample 6				
Sample 7				
Sample 8				
Sample 9				
Sample 10				
Sample 11				
Sample 12				
Ladder		✔	All Other Samples	

Chip Lot #	Reagent Kit Lot #
	2302

Chip Comments :

© Copyright 2003 - 2018 Agilent, Inc.

Assay Class: Eukaryote Total RNA Nano Created: 21-Jan-26 3:05:37 PM
Data Path: C:\...Eukaryote Total RNA Nano_DE72901994_2026-01-21_15-05-37.xad Modified: 21-Jan-26 5:35:29 PM

Electrophoresis Assay Details

General Analysis Settings

Number of Available Sample and Ladder Wells (Max.) : 13
Minimum Visible Range [s] : 17
Maximum Visible Range [s] : 70
Start Analysis Time Range [s] : 19
End Analysis Time Range [s] : 69
Ladder Concentration [ng/µl] : 150
Lower Marker Concentration [ng/µl] : 0
Upper Marker Concentration [ng/µl] : 0
Used Lower Marker for Quantitation
Standard Curve Fit is Logarithmic
Show Data Aligned to Lower Marker

Integrator Settings

Integration Start Time [s] : 19
Integration End Time [s] : 69
Slope Threshold : 0.6
Height Threshold [FU] : 0.5
Area Threshold : 0.2
Width Threshold [s] : 0.5
Baseline Plateau [s] : 6

Filter Settings

Filter Width [s] : 0.5
Polynomial Order : 4

Ladder

Ladder Peak	Size
1	25
2	200
3	500
4	1000
5	2000
6	4000

 © Copyright 2003 - 2018 Agilent, Inc.

Assay Class: Eukaryote Total RNA Nano
Data Path: C:\...Eukaryote Total RNA Nano_DE72901994_2026-01-21_15-05-37.xad
Created: 21-Jan-26 3:05:37 PM
Modified: 21-Jan-26 5:35:29 PM

Electropherogram Summary

Ladder

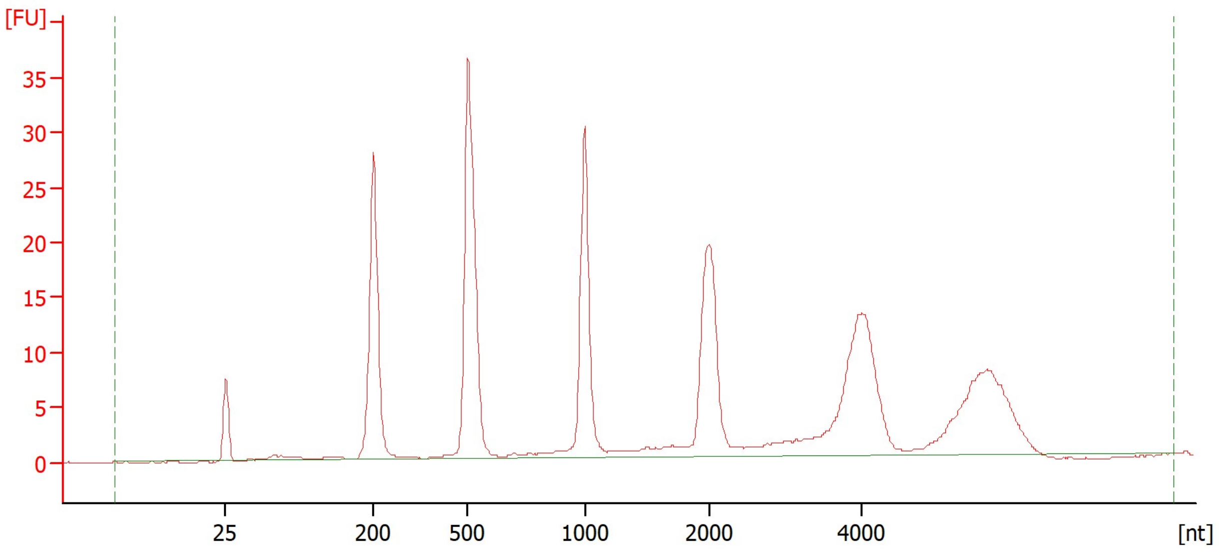

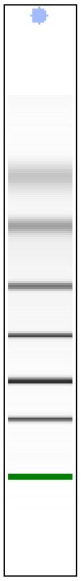

Overall Results for Ladder

RNA Area:	267.2	Result Flagging Color:	
RNA Concentration:	150 ng/µl	Result Flagging Label:	All Other Samples

© Copyright 2003 - 2018 Agilent, Inc.

Assay Class: Eukaryote Total RNA Nano
Data Path: C:\...Eukaryote Total RNA Nano_DE72901994_2026-01-21_15-05-37.xad

Created: 21-Jan-26 3:05:37 PM
Modified: 21-Jan-26 5:35:29 PM

Electropherogram Summary Continued ...

Romy AG Förstemann 1 origRNA

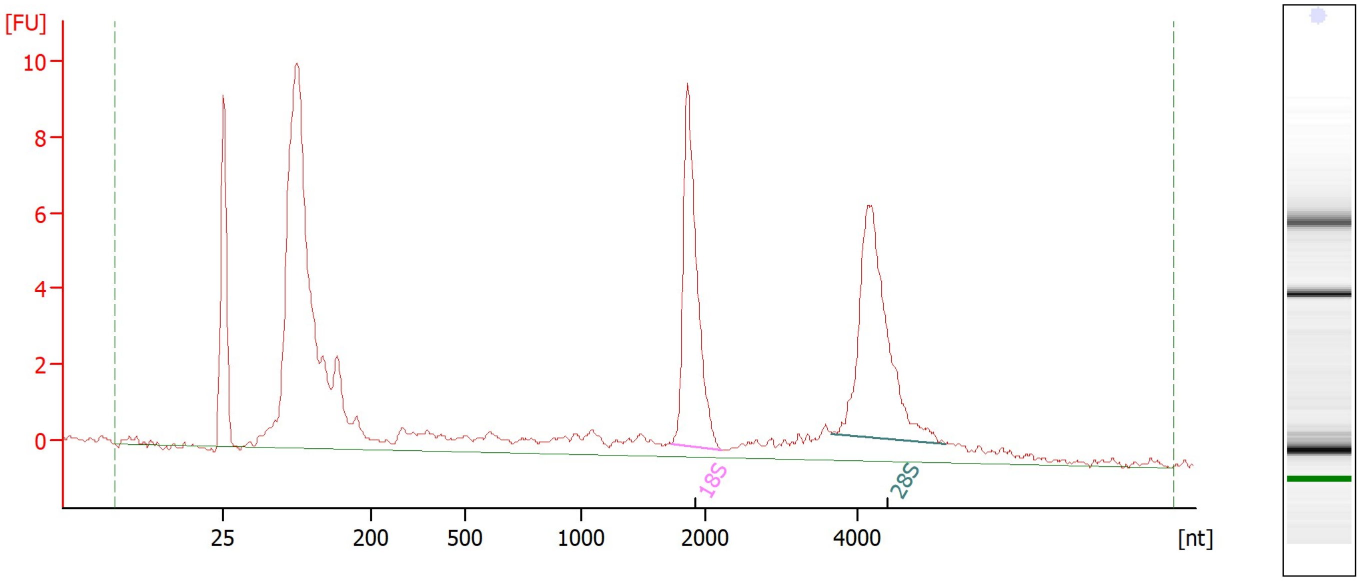

Overall Results for sample 1 : Romy AG Förstemann 1 origRNA

RNA Area:	111.6	RNA Integrity Number (RIN):	8.6 (B.02.10, Anomaly Threshold(s) manually adapted)
RNA Concentration:	63 ng/µl		
rRNA Ratio [28s / 18s]:	1.1	Result Flagging Color:	
		Result Flagging Label:	RIN: 8.60

Fragment table for sample 1 : Romy AG Förstemann 1 origRNA

Name	Start Size [nt]	End Size [nt]	Area	% of total Area
18S	1,730	2,220	15.1	13.6
28S	3,671	5,149	17.0	15.2

© Copyright 2003 - 2018 Agilent, Inc.

Assay Class:	Eukaryote Total RNA Nano	Created:	21-Jan-26 3:05:37 PM
Data Path:	C:\...Eukaryote Total RNA Nano_DE72901994_2026-01-21_15-05-37.xad	Modified:	21-Jan-26 5:35:29 PM

Electropherogram Summary Continued ...

Romy AG Förstemann 2 origRNA

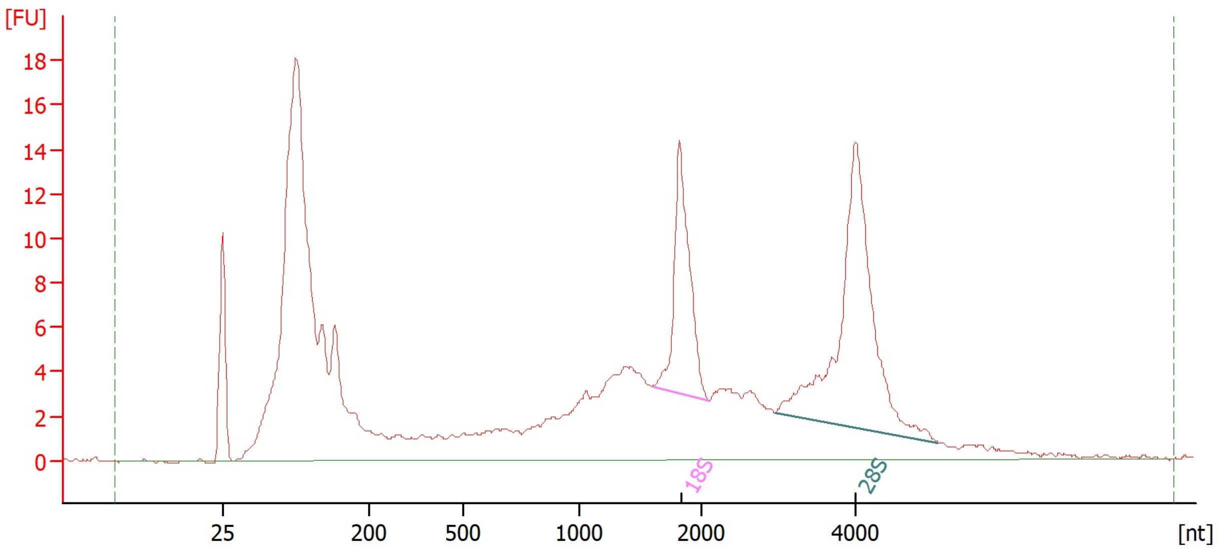

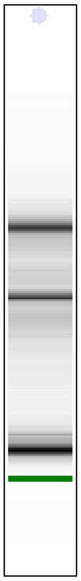

Overall Results for sample 2 : Romy AG Förstemann 2 origRNA

RNA Area:	300.2	RNA Integrity Number (RIN):	7.3 (B.02.10)
RNA Concentration:	169 ng/µl	Result Flagging Color:	
rRNA Ratio [28s / 18s]:	2.1	Result Flagging Label:	RIN: 7.30

Fragment table for sample 2 : Romy AG Förstemann 2 origRNA

Name	Start Size [nt]	End Size [nt]	Area	% of total Area
18S	1,607	2,080	19.9	6.6
28S	2,946	5,084	41.3	13.8

 © Copyright 2003 - 2018 Agilent, Inc.

Assay Class: Eukaryote Total RNA Nano
Data Path: C:\...Eukaryote Total RNA Nano_DE72901994_2026-01-21_15-05-37.xad

Created: 21-Jan-26 3:05:37 PM
Modified: 21-Jan-26 5:35:29 PM

Electropherogram Summary Continued ...

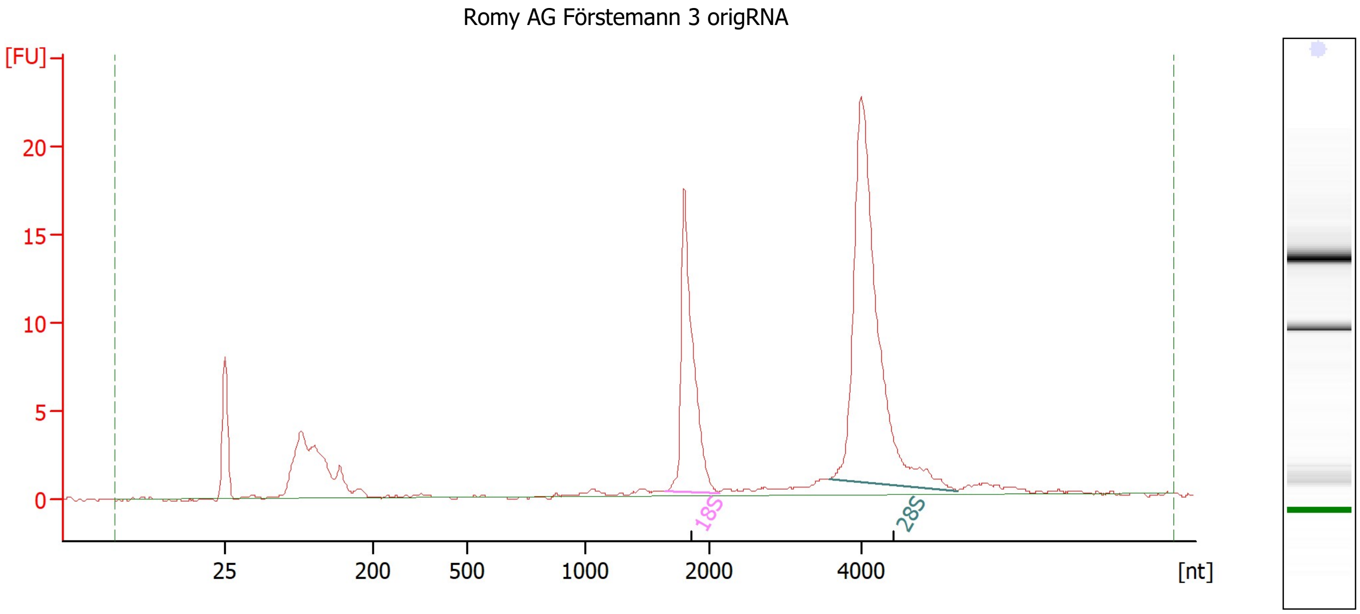

Overall Results for sample 3 : Romy AG Förstemann 3 origRNA

RNA Area:	118.4	RNA Integrity Number (RIN):	10 (B.02.10, Anomaly Threshold(s) manually adapted)
RNA Concentration:	66 ng/µl		
rRNA Ratio [28s / 18s]:	2.1	Result Flagging Color:	
		Result Flagging Label:	RIN:10

Fragment table for sample 3 : Romy AG Förstemann 3 origRNA

Name	Start Size [nt]	End Size [nt]	Area	% of total Area
18S	1,655	2,138	23.6	19.9
28S	3,583	5,236	49.9	42.1

© Copyright 2003 - 2018 Agilent, Inc.

Assay Class: Eukaryote Total RNA Nano
Data Path: C:\...Eukaryote Total RNA Nano_DE72901994_2026-01-21_15-05-37.xad
Created: 21-Jan-26 3:05:37 PM
Modified: 21-Jan-26 5:35:29 PM

Electropherogram Summary Continued ...

Romy AG Förstemann 4 origRNA

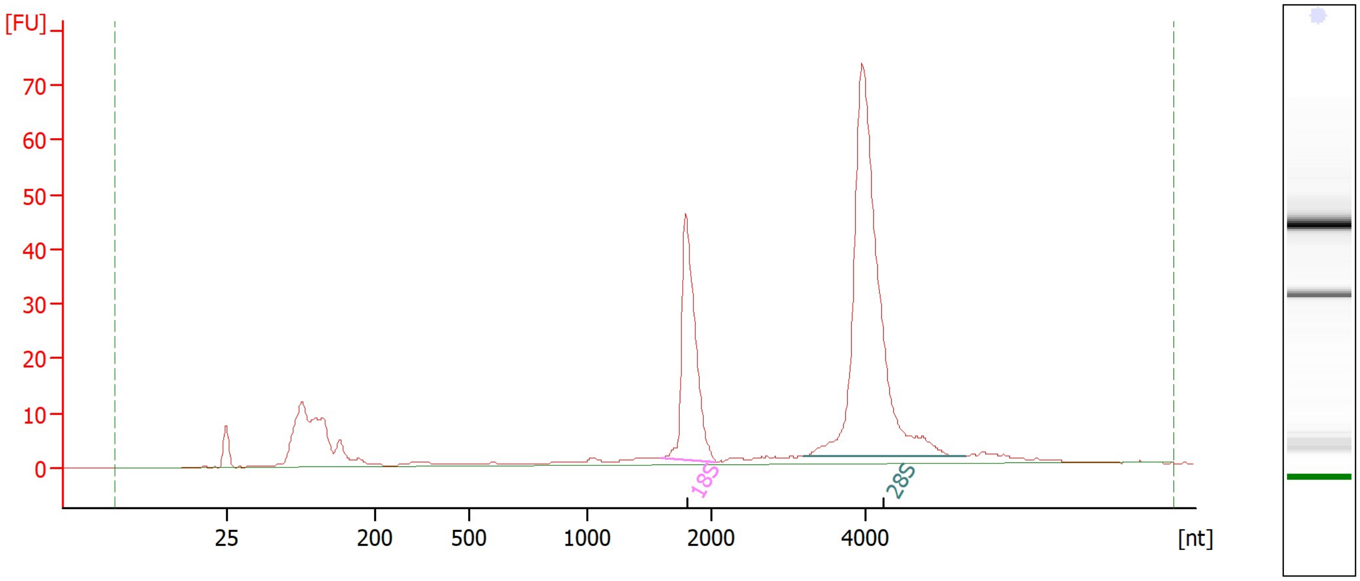

Overall Results for sample 4 : Romy AG Förstemann 4 origRNA

RNA Area:	383.3
RNA Concentration:	215 ng/µl
rRNA Ratio [28s / 18s]:	2.5
RNA Integrity Number (RIN):	10 (B.02.10, Anomaly Threshold(s) manually adapted)
Result Flagging Color:	
Result Flagging Label:	RIN:10

Fragment table for sample 4 : Romy AG Förstemann 4 origRNA

Name	Start Size [nt]	End Size [nt]	Area	% of total Area
18S	1,604	2,070	70.9	18.5
28S	3,193	5,300	174.9	45.6

© Copyright 2003 - 2018 Agilent, Inc.

Assay Class: Eukaryote Total RNA Nano
Data Path: C:\...Eukaryote Total RNA Nano_DE72901994_2026-01-21_15-05-37.xad

Created: 21-Jan-26 3:05:37 PM
Modified: 21-Jan-26 5:35:29 PM

Gel Image

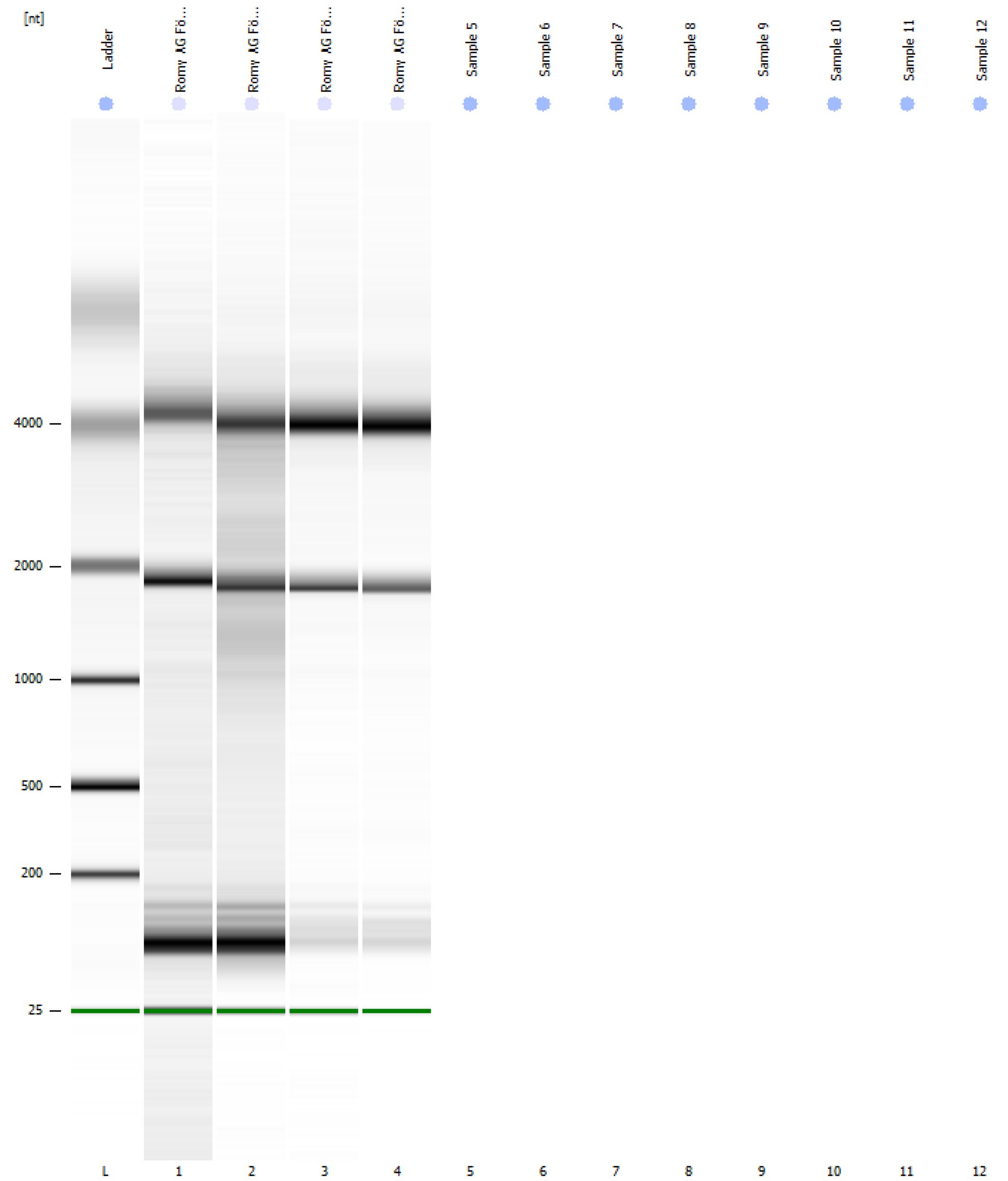

 © Copyright 2003 - 2018 Agilent, Inc.

Assay Class:	Eukaryote Total RNA Nano	Created:	21-Jan-26 3:05:37 PM
Data Path:	C:\...Eukaryote Total RNA Nano_DE72901994_2026-01-21_15-05-37.xad	Modified:	21-Jan-26 5:35:29 PM

Curves

Standard Curve

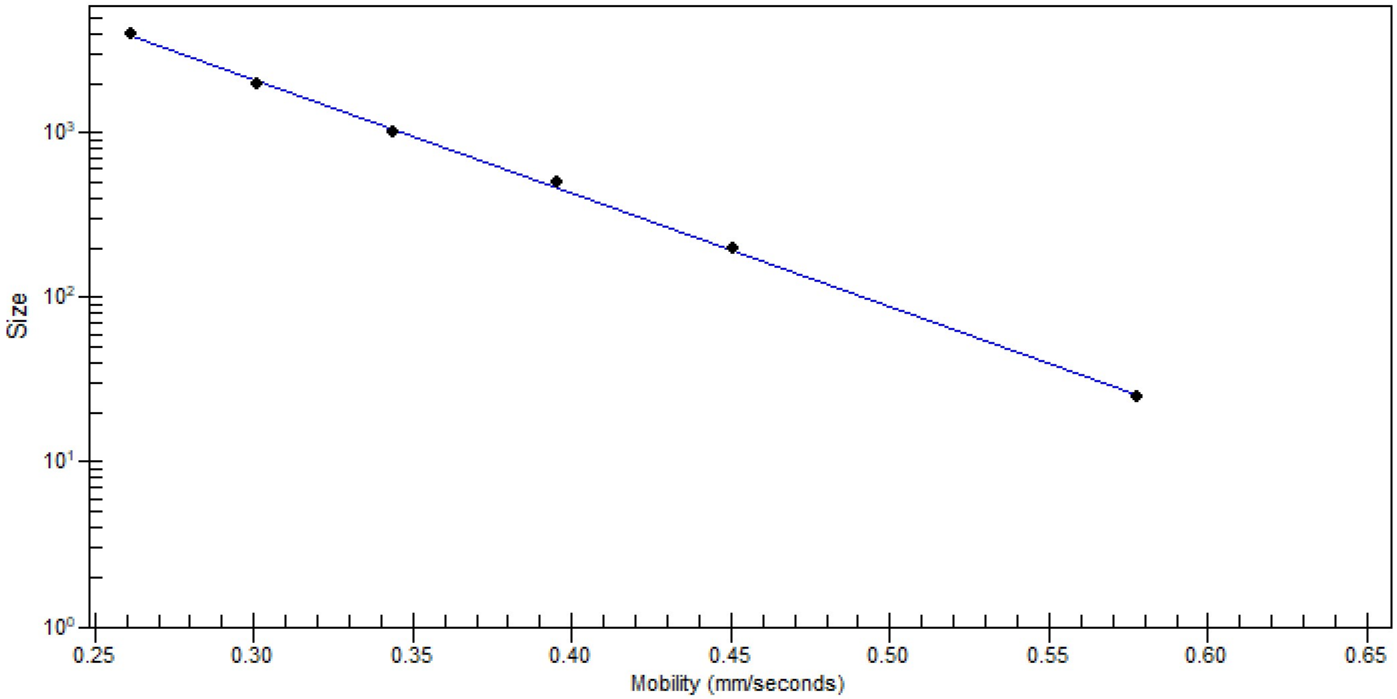

© Copyright 2003 - 2018 Agilent, Inc.
